# Predictive value of prostate-specific antigen for prostate cancer: a nested case-control study in EuroSIDA

**DOI:** 10.7448/IAS.17.4.19510

**Published:** 2014-11-02

**Authors:** Leah Shepherd, Álvaro Humberto Borges, Lene Ravn, Richard Harvey, Jean-Paul Viard, Mark Bower, Andrew Grulich, Michael Silverberg, Stephane De Wit, Ole Kirk, Jens Lundgren, Amanda Mocroft, on behalf of EuroSIDA in Eurocoord

**Affiliations:** 1 Department of Infection and Population Health, University College London, London, UK; 2 Centre for Health & Infectious Diseases Research, Rigshospitalet, University of Copenhagen, Copenhagen, Denmark; 3 Department of Clinical Biochemistry, Rigshospitalet, Copenhagen, Denmark; 4 Charing Cross Oncology Laboratory and Trophoblast, Charing Cross Hospital Campus of Imperial College Healthcare National Health Service Trust, London, UK; 5 Centre de Diagnostic et de Thérapeutique, Université Paris Descartes, Hôtel-D, Paris, France; 6 National Centre for HIV Malignancy, Chelsea and Westminster Hospital NHS Foundation Trust, London, UK; 7 Kirby Institute, The University of New South Wales, Sydney, Australia; 8 Division of Research, Kaiser Permanente Northern California, Oakland, USA; 9 Department of Infectious Diseases, Saint-Pierre Hospital, Brussels, Belgium

## Abstract

**Introduction:**

Although prostate cancer (PCa) incidence is lower in HIV+ men than in HIV− men, the usefulness of prostate-specific antigen (PSA) screening in this population is not well defined and may have higher false negative rates than in HIV− men. We aimed to describe the kinetics and predictive value of PSA in HIV+ men.

**Methods:**

Men with PCa (*n*=21) and up to two matched controls (*n*=40) with prospectively stored plasma samples before PCa (or matched date in controls) were selected. Cases and controls were matched on date of first and last sample, age, region of residence and CD4 count at first sample date. Total PSA (tPSA), free PSA (fPSA), testosterone and sex hormone binding globulin (SHBG) were measured. Conditional logistic regression models investigated associations between markers and PCa. Sensitivity and specificity of using tPSA >4 µg/L to predict PCa was calculated. Mixed models were used to describe kinetics.

**Results:**

Sixty-one men were included with a median six (IQR 2–9) years follow-up. Time between last sample and PCa was seven (4–11) months. Cases and controls were well matched at first sample, with a median age of 51 (IQR 48–57) and CD4 of 437 (243–610) cells/mm^3^. Median tPSA [2.8 (IQR: 1.6–4.6) and 0.8 (0.5–1.2) µg/L] and fPSA [0.4 (0.2–0.8) and 0.3 (0.2–0.4) µg/L] levels were higher in cases than controls at first sample. Both tPSA and fPSA increased significantly over time in cases (Figure 1), to a median at last sample of 6.1 (4.7–9.5) and 0.9 (0.6–1.3) µg/L, respectively, but were stable in controls, with a median at last sample of 0.8 (0.5–1.4) and 0.2 (0.2–0.4) µg/L (Figure). Higher levels of tPSA and fPSA were associated with higher odds of PCa at first sample [OR for 2-fold higher 4.7 (CI: 1.7–12.9) and 5.4 (1.7–17.4)]. Elevated tPSA values in cases were detectable ≥5 years before PCa (*p*<0.01). Testosterone [overall median 19.4 (IQR 15.3–23.9) nmol/L at first sample) and SHBG [50.0 (34.0–66.0) nmol/L] levels were similar in cases and controls at first and last sample (all *p*>0.7). The most informative predictor of PCa was tPSA (AUC=0.9), followed by fPSA (0.8). Testosterone (AUC = 0.5) and SHBG (0.5) were poor predictors of PCa. Overall, tPSA level >4 µg/L had 99% specificity and 37% sensitivity. Performance was best in the year prior to PCa (specificity: 99%, sensitivity: 88%).

**Conclusions:**

PSA was highly predictive of PCa in HIV+ men. Our results indicate that PSA screening in HIV+ men may be useful, and further work is needed to identify potentially age-related cut-offs to maximize sensitivity and specificity to identify those for further evaluation at early stages of PCa.

**Figure 1:**
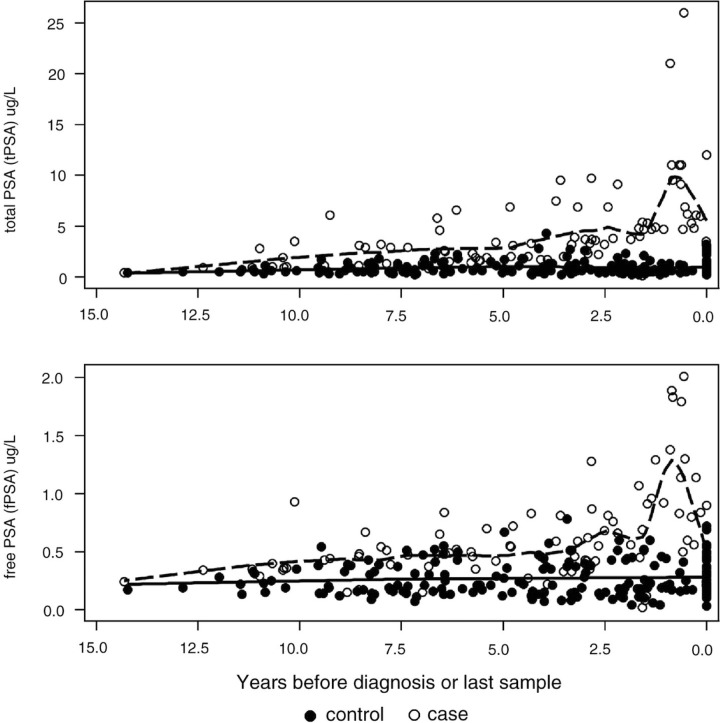
Total PSA (tPSA) and free PSA (fPSA) levels by time before diagnosis (or last sample date in controls) with superimposed loess curves. tPSA and fPSA levels were increasing by 13.7 (10.3, 17.3)% and 7.9 (4.7, 11.2)% per year since baseline in cases (both *p*<0.01) and were stable in controls (*p*=0.67 and 0.36). Figure includes multiple measurements per man.

